# Good News for the Nation

**Published:** 1921-02-19

**Authors:** 


### Good News for the Nation.

We do not wish to draw unfair or fantastic inferences from the provisional figures of the Registrar-General for England and Wales of the births and deaths for 1920. But it is at least instructive to observe that at a time when any stick is good enough to beat the Health Ministry, and when an insidious campaign is on foot to suggest the futility of State action in matters of public health, the death-rate and infant-mortality rate are the lowest ever recorded. The general death-rate is now down to 12.4 per 1,000 of the population, compared with 24 fifty years ago, and the total number of deaths is actually no more than it was in 1862, when the whole population was only 20,000,000. Even more striking is the decrease in infant mortality, which stands at 80 per 1,000 births, compared with 97 in 1916, 128 from 1901 to 1910, and 153 from 1891 to 1900. What do these figures mean? If they mean anything, they mean that the improved methods of public sanitation and hygiene, the greater care in the notification of disease, the extension of early medical treatment through the panel system, and the closer grip which the State is exercising in every direction are now beginning to show substantial results. Particularly in the case of the remarkable saving in child life is the protection and guidance of the State a proved factor. Here it is not so much the advance of medical science as the advance of education—through what has been called the Baby Welfare Service, through the establishment of health centres and other powerful agencies—which is removing the old reproach of the wastage of infant life; and the backbone of this beneficent work is the timely "interference" of the State in every stage of child-life, in infancy and throughout the school existence. The figures must give great encouragement to harassed health officials and the fast-growing army of social workers who have thrown themselves heart and soul into the task of health reform; and we note with satisfaction that the statistics seem, momentarily at any rate, to have silenced those croakers in the Press who have been predicting disaster through the wider stretch of Government control.

On the other hand, the rising birth-rate, the highest of the decade, which is a hopeful symptom of race recovery at the other end of the scale, was anticipated only by a few experts, and certainly the significance of the rise has excited general surprise. We have no space to examine all the contributing causes. The check after the war to the normal flow of emigration may be one of them; it is also well known that during prosperous times population tends to increase substantially, and up to the middle of last summer the post-war condition of Great Britain certainly bore every evidence of prosperity. The one regrettable feature of the rising birth-rate is that, as far as general observation goes, it applies less to the middle class than to any other, and the middle class is perhaps the one considerable element in the population which did not share in the higher standard of living which immediately followed the war period. There are undoubtedly numerous sociological experts who regard these figures as showing an unfortunate tendency. They are of the school which argues that an unchecked growth in the population of the world will ultimately destroy the world, and which therefore seeks strongly to encourage the development of an enlightened and general system of birth-control. Recently we quoted the views of an American doctor, who holds that only by a rigorous regulation of births can another world war be avoided, because, so the familiar theory runs, the underlying cause of the last war was the necessity of obtaining more land and more food for over-populated countries. It is an attractive theory, but, for this country in particular, it is a dangerous one. We are an industrial nation. Industrialism is the very core of our existence, and the breath of its life is a large and vigorous population. To check its natural growth on a large scale by artificial means would be to exercise a weapon of unknown and incalculable power. The effect, if it were to diminish our numbers below a certain limit without any assurance of our ability to recover, would be to destroy the industrialism which is the source of our wealth, and possibly to reduce us to the status of a fifth-rate country, dependent largely upon agriculture for its livelihood, and condemned to a standard of living much lower than that of the eighteenth century. Nor should we pass to that condition without an intervening period of revolutionary misery and chaos. There is much to be thankful for in a rising birth-rate, as there is in a falling death-rate.

